# Pecha Kucha presentations by medical students in Spain

**DOI:** 10.5116/ijme.5b92.52e3

**Published:** 2018-09-19

**Authors:** José Manuel Ramos-Rincón, Teresa Sempere-Selva Sempere-Selva, Mónica Romero-Nieto, Jorge Peris-García, Guillermo Martínez-de la Torre, Meggan Harris, Javier Fernández-Sánchez

**Affiliations:** 1 Department of Clinical Medicine, School of Medicine, Sant Joan d Alacant Campus, Miguel Hernandez University of Elche, Spain; 2 Innovation and Teaching Support Service, Elche Campus, Miguel Hernandez University of Elche, Spain; 3 Independent researcher, Valencia, Spain

**Keywords:** Pecha Kucha, presentations, medical students, Spain

## To the Editor

Presenting scientific information is an important skill for transmitting knowledge to the public, and it often takes the form of PowerPoint presentations.^1^ However, speakers who have not developed good communication skills, including young and undergraduate students,^1^^,^^2^ tend to overuse slides and exceed time limitations.^3^

In order to provide our medical students with opportunities to develop their concision and creativity^2^^,^^3^—skills that are valued in the workforce as well is in other interpersonal contexts^2^^-^^4^^,^^5^ —we piloted a unit on Pecha Kucha presentations into Integrated Workshops II, a third-year practical course at the School of Medicine of Miguel Hernandez University, in Elche (Alicante), Spain.  The name Pecha Kucha comes from the Japanese pa-chok-cha, meaning chit-chat, and it refers to a presentation method developed by Astrid Klein and Mark Dytham in Tokyo in 2003 to sustain the audience’s interest and attention over a series of different presentations. Information is simply and informally structured into 20 slides, shown for 20 seconds each, for a total presentation time of 6 minutes, 40 seconds.^6^ Requiring conscientious editing, rigorous preparation and practice, and critical communication skills, this “20 × 20” format aims to create dynamic and systematic presentations of all relevant information worth sharing with the audience, making it an innovative and valuable tool in the context of university education.^4^^,^^7^^,^^8^

While Pecha Kucha has been applied in educational contexts in odontology,^3^ anthropology,^9^ psychology,^10 ^business and marketing,^5^ there are only a few examples in medical or nursing education.^2^^,^^7^^,^^8^ During the course, our students put together 30 Pecha Kucha presentations in teams of four, on topics like complementary tests, examinations and special techniques in clinical medicine. Two Pecha Kucha Kucha events were held near the end of the term (May 9 and 11, 2017), each with 15 presentations. Students gave feedback on their classmates’ work after each event, and three weeks afterward, they completed a satisfaction survey on the experience.  Student audience members assigned a mean score of 8.5 over 10 (range 7.7 to 9.1) to their classmates’ interventions, and a mean score of 8 over 10 for their overall satisfaction with the Pecha Kucha unit.

Following this successful experience, Pecha Kucha presentations have been incorporated as a regular teaching aid in the School of Medicine. We believe that this format is suitable for application in other health sciences degrees and disciplines of medical education, both by instructors giving lectures and by students presenting research and class assignments. In that sense, we would encourage teachers in medical education to consider using this tool and sharing their experiences in settings for applied educational innovation. In short, Pecha Kucha has the potential to foster students’ capacities in abstraction, analysis, and synthesis.

### Acknowledgments

We are also grateful to the Associate Vice Rector for Technological Innovation, Maria Asunción Martinez Mayoral, for her unconditional support to the faculty, which greatly encouraged the performance of the project.

This project was supported by a grant from the PIEU-UMH Program for Innovative University Education 2016-2017 (PIEU2016/22). Vicerectorship on Research and Innovation, Miguel Hernández University of Elche.

### Conflict of Interest

No potential conflict of interest was reported by the authors.
